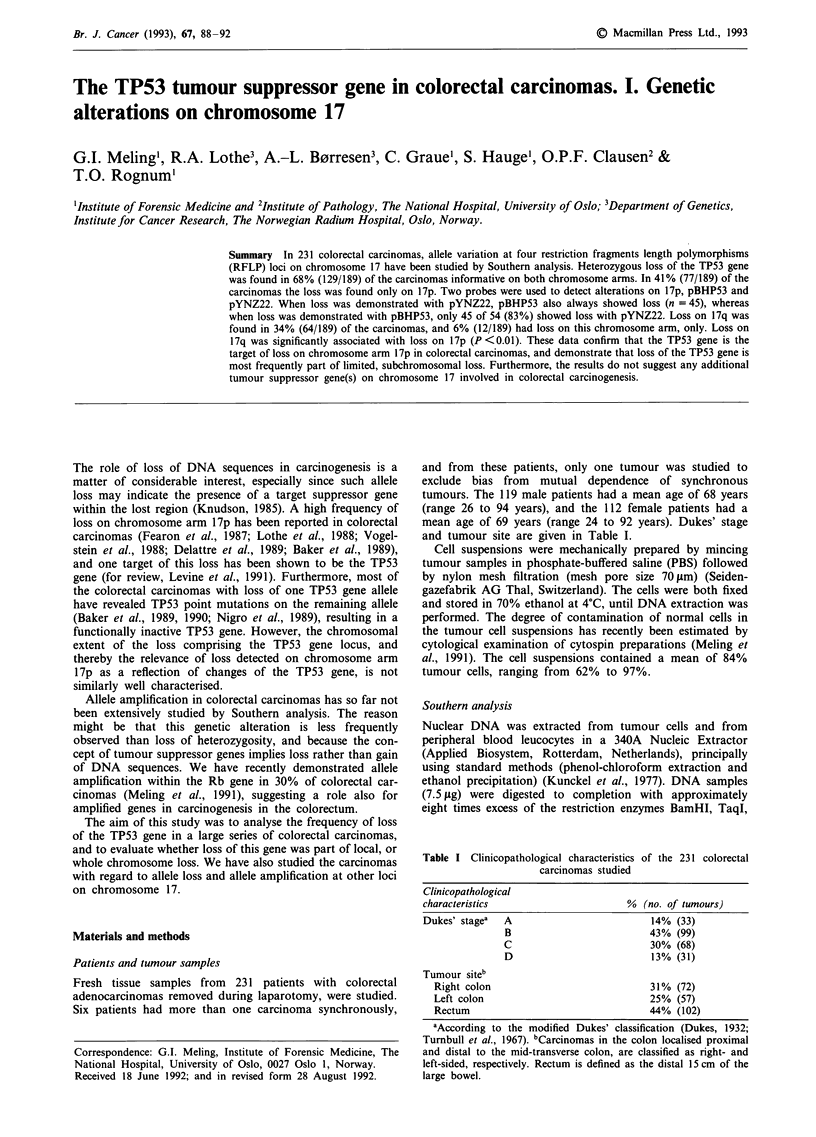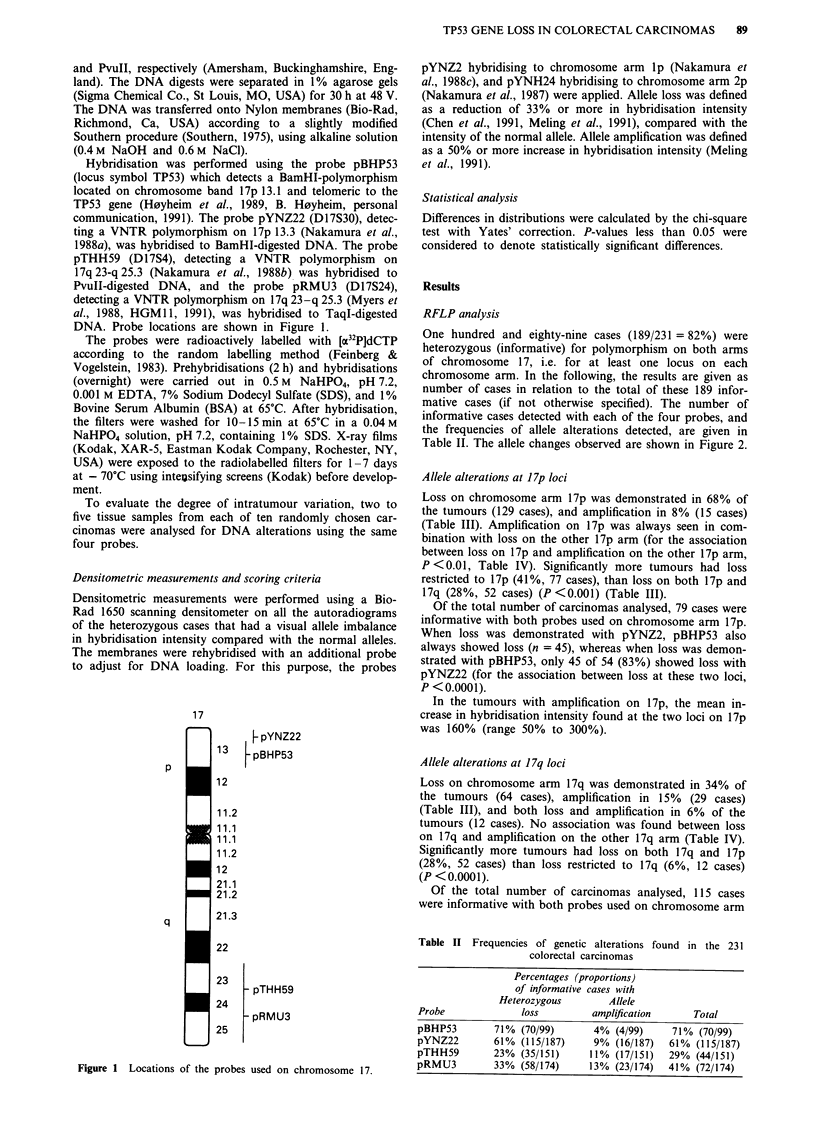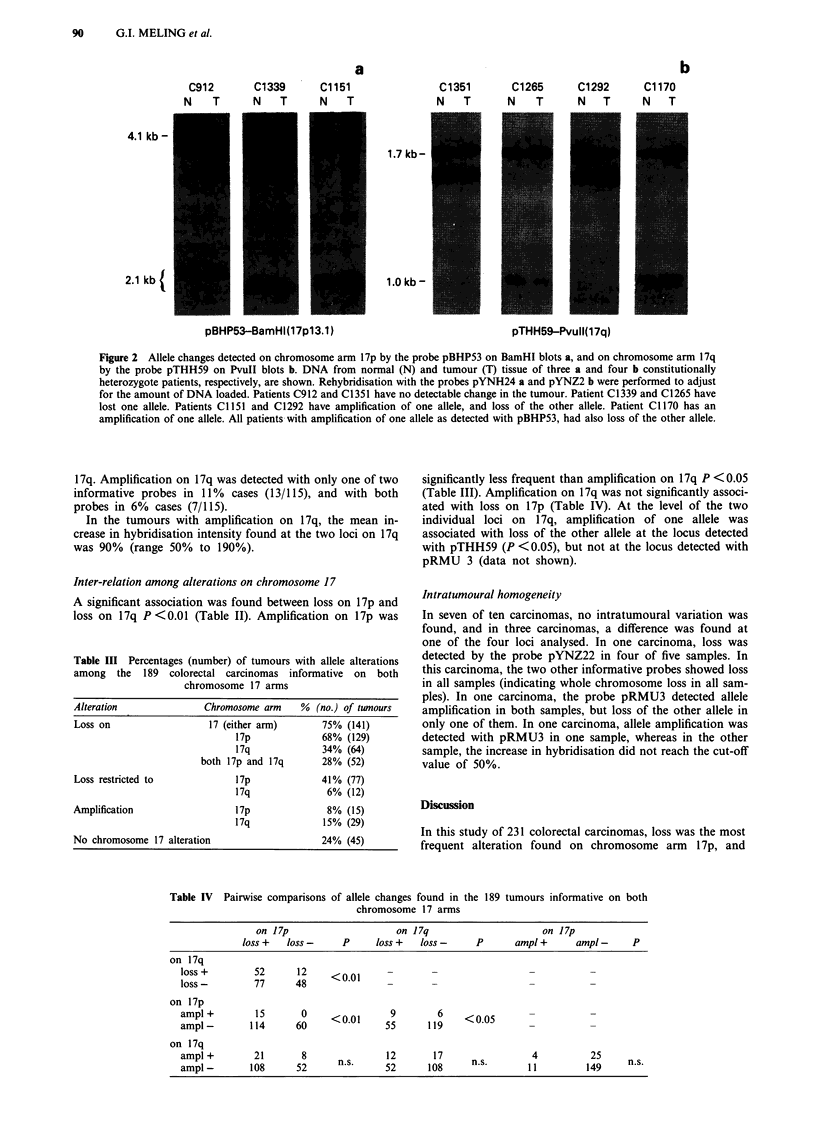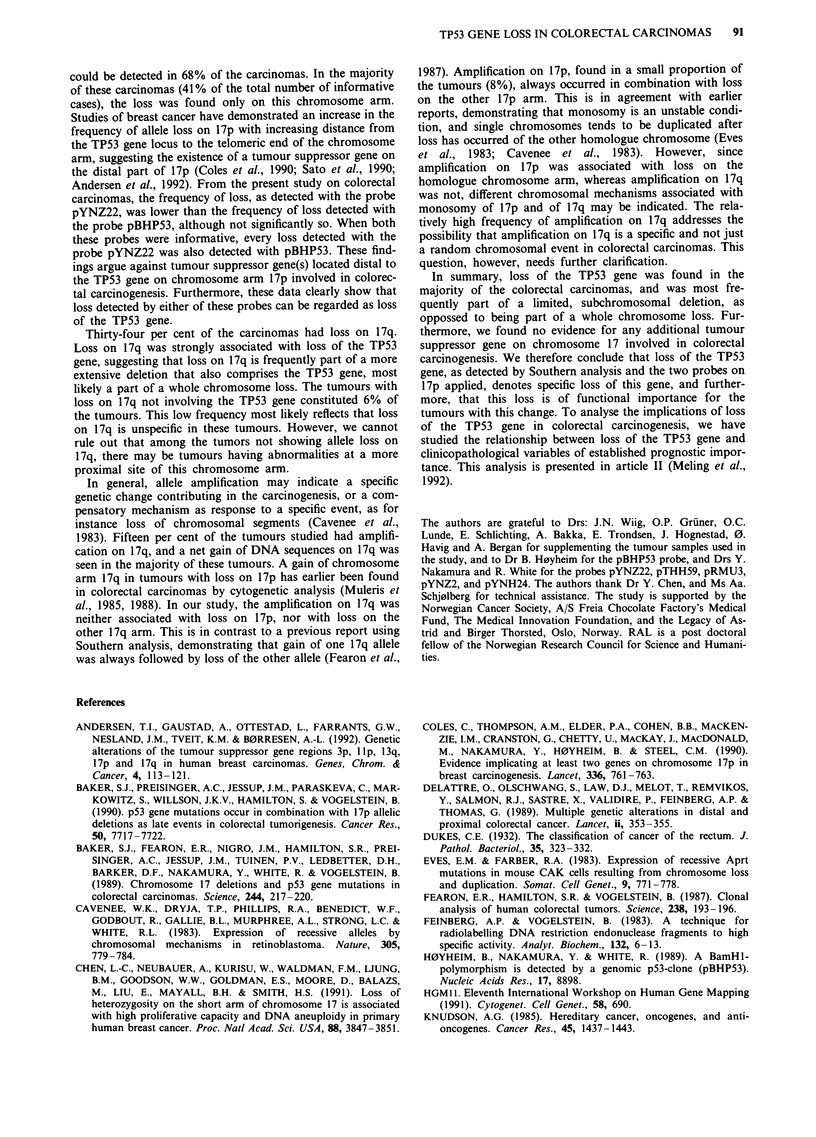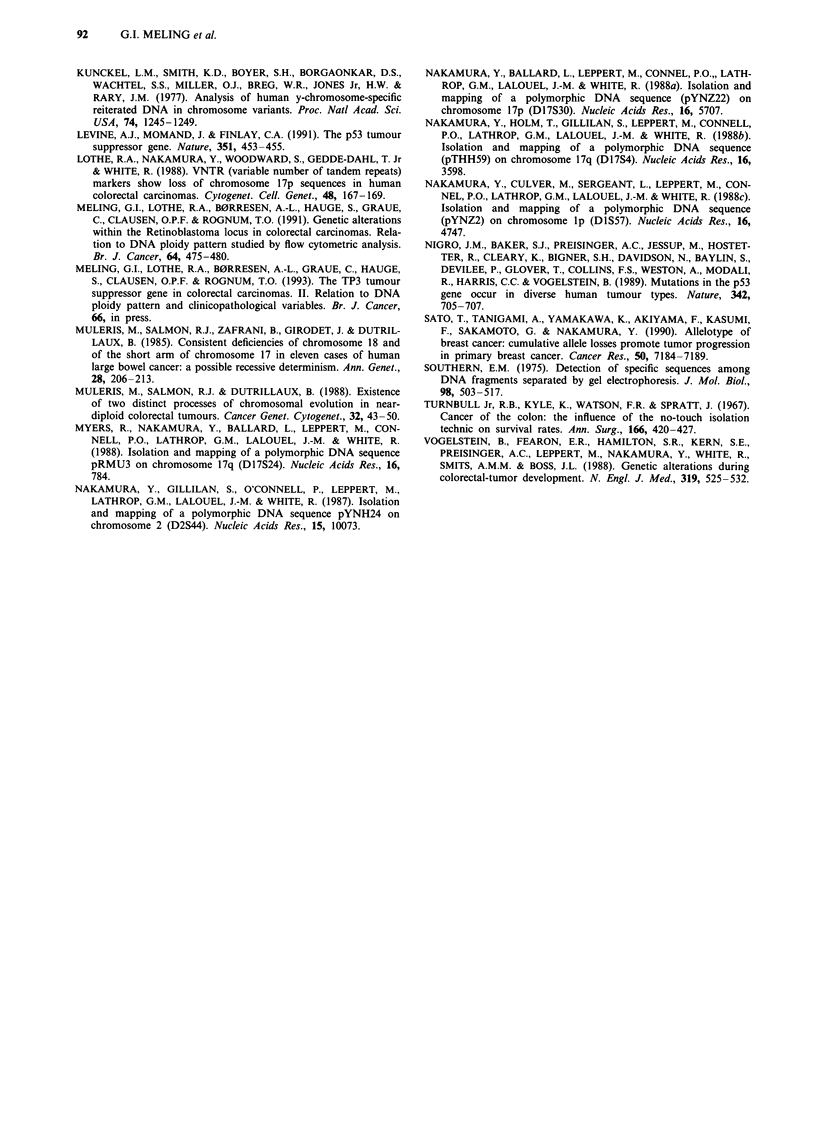# The TP53 tumour suppressor gene in colorectal carcinomas. I. Genetic alterations on chromosome 17.

**DOI:** 10.1038/bjc.1993.14

**Published:** 1993-01

**Authors:** G. I. Meling, R. A. Lothe, A. L. Børresen, C. Graue, S. Hauge, O. P. Clausen, T. O. Rognum

**Affiliations:** Institute of Forensic Medicine, National Hospital, University of Oslo, Norway.

## Abstract

**Images:**


Br. J. Cancer (1993), 67, 88–92 © Macmillan Press Ltd., 1993

### The TP53 tumour suppressor gene in colorectal carcinomas. I. Genetic alterations on chromosome 17

G.I. Meling[1], R.A. Lothe[3], A.–L. Børresen[3], C. Graue[1], S. Hauge[1], O.P.F. Clausen[2] & T.O. Rognum[1]

[1]*Institute of Forensic Medicine and* [2]*Institute of Pathology, The National Hospital, University of Oslo;* [3]*Department of Genetics, Institute for Cancer Research, The Norwegian Radium Hospital, Oslo, Norway.*

**Summary** In 231 colorectal carcinomas, allele variation at four restriction fragments length polymorphisms (RFLP) loci on chromosome 17 have been studied by Southern analysis. Heterozygous loss of the TP53 gene was found in 68% (129/189) of the carcinomas informative on both chromosome arms. In 41% (77/189) of the carcinomas the loss was found only on 17p. Two probes were used to detect alterations on 17p, pBHP53 and pYNZ22. When loss was demonstrated with pYNZ22, pBHP53 also always showed loss ($n$ = 45), whereas when loss was demonstrated with pBHP53, only 45 of 54 (83%) showed loss with pYNZ22. Loss on 17q was found in 34% (64/189) of the carcinomas, and 6% (12/189) had loss on this chromosome arm, only. Loss on 17q was significantly associated with loss on 17p ($P < 0.01$). These data confirm that the TP53 gene is the target of loss on chromosome arm 17p in colorectal carcinomas, and demonstrate that loss of the TP53 gene is most frequently part of limited, subchromosomal loss. Furthermore, the results do not suggest any additional tumour suppressor gene(s) on chromosome 17 involved in colorectal carcinogenesis.

The role of loss of DNA sequences in carcinogenesis is a matter of considerable interest, especially since such allele loss may indicate the presence of a target suppressor gene within the lost region (Knudson, 1985). A high frequency of loss on chromosome arm 17p has been reported in colorectal carcinomas (Fearon *et al.*, 1987; Lothe *et al.*, 1988; Vogelstein *et al.*, 1988; Delattre *et al.*, 1989; Baker *et al.*, 1989), and one target of this loss has been shown to be the TP53 gene (for review, Levine *et al.*, 1991). Furthermore, most of the colorectal carcinomas with loss of one TP53 gene allele have revealed TP53 point mutations on the remaining allele (Baker *et al.*, 1989, 1990; Nigro *et al.*, 1989), resulting in a functionally inactive TP53 gene. However, the chromosomal extent of the loss comprising the TP53 gene locus, and thereby the relevance of loss detected on chromosome arm 17p as a reflection of changes of the TP53 gene, is not similarly well characterised.

Allele amplification in colorectal carcinomas has so far not been extensively studied by Southern analysis. The reason might be that this genetic alteration is less frequently observed than loss of heterozygosity, and because the concept of tumour suppressor genes implies loss rather than gain of DNA sequences. We have recently demonstrated allele amplification within the Rb gene in 30% of colorectal carcinomas (Meling *et al.*, 1991), suggesting a role also for amplified genes in carcinogenesis in the colorectum.

The aim of this study was to analyse the frequency of loss of the TP53 gene in a large series of colorectal carcinomas, and to evaluate whether loss of this gene was part of local, or whole chromosome loss. We have also studied the carcinomas with regard to allele loss and allele amplification at other loci on chromosome 17.

### Materials and methods

### *Patients and tumour samples*

Fresh tissue samples from 231 patients with colorectal adenocarcinomas removed during laparotomy, were studied. Six patients had more than one carcinoma synchronously, and from these patients, only one tumour was studied to exclude bias from mutual dependence of synchronous tumours. The 119 male patients had a mean age of 68 years (range 26 to 94 years), and the 112 female patients had a mean age of 69 years (range 24 to 92 years). Dukes' stage and tumour site are given in Table I.

Cell suspensions were mechanically prepared by mincing tumour samples in phosphate-buffered saline (PBS) followed by nylon mesh filtration (mesh pore size 70 µm) (Seidengazefabrik AG Thal, Switzerland). The cells were both fixed and stored in 70% ethanol at 4°C, until DNA extraction was performed. The degree of contamination of normal cells in the tumour cell suspensions has recently been estimated by cytological examination of cytospin preparations (Meling *et al.*, 1991). The cell suspensions contained a mean of 84% tumour cells, ranging from 62% to 97%.

### *Southern analysis*

Nuclear DNA was extracted from tumour cells and from peripheral blood leucocytes in a 340A Nucleic Extractor (Applied Biosystem, Rotterdam, Netherlands), principally using standard methods (phenol-chloroform extraction and ethanol precipitation) (Kunckel *et al.*, 1977). DNA samples (7.5 µg) were digested to completion with approximately eight times excess of the restriction enzymes BamHI, TaqI,

**Table I** Clinicopathological characteristics of the 231 colorectal carcinomas studied

| *Clinicopathological characteristics* | | *% (no. of tumours)* |
|---|---|---|
| Dukes' stage[a] | A | 14% (33) |
| | B | 43% (99) |
| | C | 30% (68) |
| | D | 13% (31) |
| Tumour site[b] | | |
| Right colon | | 31% (72) |
| Left colon | | 25% (57) |
| Rectum | | 44% (102) |

[a]According to the modified Dukes' classification (Dukes, 1932; Turnbull *et al.*, 1967). [b]Carcinomas in the colon localised proximal and distal to the mid-transverse colon, are classified as right- and left-sided, respectively. Rectum is defined as the distal 15 cm of the large bowel.

Correspondence: G.I. Meling, Institute of Forensic Medicine, The National Hospital, University of Oslo, 0027 Oslo 1, Norway.
Received 18 June 1992; and in revised form 28 August 1992.

and PvuII, respectively (Amersham, Buckinghamshire, England). The DNA digests were separated in 1% agarose gels (Sigma Chemical Co., St Louis, MO, USA) for 30 h at 48 V. The DNA was transferred onto Nylon membranes (Bio-Rad, Richmond, Ca, USA) according to a slightly modified Southern procedure (Southern, 1975), using alkaline solution (0.4 M NaOH and 0.6 M NaCl).

Hybridisation was performed using the probe pBHP53 (locus symbol TP53) which detects a BamHI-polymorphism located on chromosome band 17p 13.1 and telomeric to the TP53 gene (Høyheim *et al.*, 1989, B. Høyheim, personal communication, 1991). The probe pYNZ22 (D17S30), detecting a VNTR polymorphism on 17p 13.3 (Nakamura *et al.*, 1988*a*), was hybridised to BamHI-digested DNA. The probe pTHH59 (D17S4), detecting a VNTR polymorphism on 17q 23-q 25.3 (Nakamura *et al.*, 1988*b*) was hybridised to PvuII-digested DNA, and the probe pRMU3 (D17S24), detecting a VNTR polymorphism on 17q 23–q 25.3 (Myers *et al.*, 1988, HGM11, 1991), was hybridised to TaqI-digested DNA. Probe locations are shown in Figure 1.

The probes were radioactively labelled with [$\alpha^{32}$P]dCTP according to the random labelling method (Feinberg & Vogelstein, 1983). Prehybridisations (2 h) and hybridisations (overnight) were carried out in 0.5 M $NaHPO_4$, pH 7.2, 0.001 M EDTA, 7% Sodium Dodecyl Sulfate (SDS), and 1% Bovine Serum Albumin (BSA) at 65°C. After hybridisation, the filters were washed for 10–15 min at 65°C in a 0.04 M $NaHPO_4$ solution, pH 7.2, containing 1% SDS. X-ray films (Kodak, XAR-5, Eastman Kodak Company, Rochester, NY, USA) were exposed to the radiolabelled filters for 1–7 days at − 70°C using intensifying screens (Kodak) before development.

To evaluate the degree of intratumour variation, two to five tissue samples from each of ten randomly chosen carcinomas were analysed for DNA alterations using the same four probes.

*Densitometric measurements and scoring criteria*

Densitometric measurements were performed using a Bio-Rad 1650 scanning densitometer on all the autoradiograms of the heterozygous cases that had a visual allele imbalance in hybridisation intensity compared with the normal alleles. The membranes were rehybridised with an additional probe to adjust for DNA loading. For this purpose, the probes

**Figure 1** Locations of the probes used on chromosome 17.

pYNZ2 hybridising to chromosome arm 1p (Nakamura *et al.*, 1988*c*), and pYNH24 hybridising to chromosome arm 2p (Nakamura *et al.*, 1987) were applied. Allele loss was defined as a reduction of 33% or more in hybridisation intensity (Chen *et al.*, 1991, Meling *et al.*, 1991), compared with the intensity of the normal allele. Allele amplification was defined as a 50% or more increase in hybridisation intensity (Meling *et al.*, 1991).

*Statistical analysis*

Differences in distributions were calculated by the chi-square test with Yates' correction. *P*-values less than 0.05 were considered to denote statistically significant differences.

### Results

*RFLP analysis*

One hundred and eighty-nine cases (189/231 = 82%) were heterozygous (informative) for polymorphism on both arms of chromosome 17, i.e. for at least one locus on each chromosome arm. In the following, the results are given as number of cases in relation to the total of these 189 informative cases (if not otherwise specified). The number of informative cases detected with each of the four probes, and the frequencies of allele alterations detected, are given in Table II. The allele changes observed are shown in Figure 2.

*Allele alterations at 17p loci*

Loss on chromosome arm 17p was demonstrated in 68% of the tumours (129 cases), and amplification in 8% (15 cases) (Table III). Amplification on 17p was always seen in combination with loss on the other 17p arm (for the association between loss on 17p and amplification on the other 17p arm, $P < 0.01$, Table IV). Significantly more tumours had loss restricted to 17p (41%, 77 cases), than loss on both 17p and 17q (28%, 52 cases) ($P < 0.001$) (Table III).

Of the total number of carcinomas analysed, 79 cases were informative with both probes used on chromosome arm 17p. When loss was demonstrated with pYNZ2, pBHP53 also always showed loss ($n = 45$), whereas when loss was demonstrated with pBHP53, only 45 of 54 (83%) showed loss with pYNZ22 (for the association between loss at these two loci, $P < 0.0001$).

In the tumours with amplification on 17p, the mean increase in hybridisation intensity found at the two loci on 17p was 160% (range 50% to 300%).

*Allele alterations at 17q loci*

Loss on chromosome arm 17q was demonstrated in 34% of the tumours (64 cases), amplification in 15% (29 cases) (Table III), and both loss and amplification in 6% of the tumours (12 cases). No association was found between loss on 17q and amplification on the other 17q arm (Table IV). Significantly more tumours had loss on both 17q and 17p (28%, 52 cases) than loss restricted to 17q (6%, 12 cases) ($P < 0.0001$).

Of the total number of carcinomas analysed, 115 cases were informative with both probes used on chromosome arm

**Table II** Frequencies of genetic alterations found in the 231 colorectal carcinomas

| *Probe* | *Percentages (proportions) of informative cases with Heterozygous loss* | *Allele amplification* | *Total* |
|---|---|---|---|
| pBHP53 | 71% (70/99) | 4% (4/99) | 71% (70/99) |
| pYNZ22 | 61% (115/187) | 9% (16/187) | 61% (115/187) |
| pTHH59 | 23% (35/151) | 11% (17/151) | 29% (44/151) |
| pRMU3 | 33% (58/174) | 13% (23/174) | 41% (72/174) |

**Figure 2** Allele changes detected on chromosome arm 17p by the probe pBHP53 on BamHI blots **a**, and on chromosome arm 17q by the probe pTHH59 on PvuII blots **b**. DNA from normal (N) and tumour (T) tissue of three **a** and four **b** constitutionally heterozygote patients, respectively, are shown. Rehybridisation with the probes pYNH24 **a** and pYNZ2 **b** were performed to adjust for the amount of DNA loaded. Patients C912 and C1351 have no detectable change in the tumour. Patient C1339 and C1265 have lost one allele. Patients C1151 and C1292 have amplification of one allele, and loss of the other allele. Patient C1170 has an amplification of one allele. All patients with amplification of one allele as detected with pBHP53, had also loss of the other allele.

17q. Amplification on 17q was detected with only one of two informative probes in 11% cases (13/115), and with both probes in 6% cases (7/115).

In the tumours with amplification on 17q, the mean increase in hybridisation intensity found at the two loci on 17q was 90% (range 50% to 190%).

*Inter-relation among alterations on chromosome 17*

A significant association was found between loss on 17p and loss on 17q $P < 0.01$ (Table II). Amplification on 17p was significantly less frequent than amplification on 17q $P < 0.05$ (Table III). Amplification on 17q was not significantly associated with loss on 17p (Table IV). At the level of the two individual loci on 17q, amplification of one allele was associated with loss of the other allele at the locus detected with pTHH59 ($P < 0.05$), but not at the locus detected with pRMU 3 (data not shown).

**Table III** Percentages (number) of tumours with allele alterations among the 189 colorectal carcinomas informative on both chromosome 17 arms

| *Alteration* | *Chromosome arm* | *% (no.) of tumours* |
|---|---|---|
| Loss on | 17 (either arm) | 75% (141) |
| | 17p | 68% (129) |
| | 17q | 34% (64) |
| | both 17p and 17q | 28% (52) |
| Loss restricted to | 17p | 41% (77) |
| | 17q | 6% (12) |
| Amplification | 17p | 8% (15) |
| | 17q | 15% (29) |
| No chromosome 17 alteration | | 24% (45) |

*Intratumoural homogeneity*

In seven of ten carcinomas, no intratumoural variation was found, and in three carcinomas, a difference was found at one of the four loci analysed. In one carcinoma, loss was detected by the probe pYNZ22 in four of five samples. In this carcinoma, the two other informative probes showed loss in all samples (indicating whole chromosome loss in all samples). In one carcinoma, the probe pRMU3 detected allele amplification in both samples, but loss of the other allele in only one of them. In one carcinoma, allele amplification was detected with pRMU3 in one sample, whereas in the other sample, the increase in hybridisation did not reach the cut-off value of 50%.

### Discussion

In this study of 231 colorectal carcinomas, loss was the most frequent alteration found on chromosome arm 17p, and

**Table IV** Pairwise comparisons of allele changes found in the 189 tumours informative on both chromosome 17 arms

| | *on 17p* | | | *on 17q* | | | *on 17p* | | |
|---|---|---|---|---|---|---|---|---|---|
| | *loss +* | *loss –* | *P* | *loss +* | *loss –* | *P* | *ampl +* | *ampl –* | *P* |
| on 17q | | | | | | | | | |
| loss + | 52 | 12 | <0.01 | – | – | | – | – | |
| loss – | 77 | 48 | | – | – | | – | – | |
| on 17p | | | | | | | | | |
| ampl + | 15 | 0 | <0.01 | 9 | 6 | <0.05 | – | – | |
| ampl – | 114 | 60 | | 55 | 119 | | – | – | |
| on 17q | | | | | | | | | |
| ampl + | 21 | 8 | n.s. | 12 | 17 | n.s. | 4 | 25 | n.s. |
| ampl – | 108 | 52 | | 52 | 108 | | 11 | 149 | |

could be detected in 68% of the carcinomas. In the majority of these carcinomas (41% of the total number of informative cases), the loss was found only on this chromosome arm. Studies of breast cancer have demonstrated an increase in the frequency of allele loss on 17p with increasing distance from the TP53 gene locus to the telomeric end of the chromosome arm, suggesting the existence of a tumour suppressor gene on the distal part of 17p (Coles *et al.*, 1990; Sato *et al.*, 1990; Andersen *et al.*, 1992). From the present study on colorectal carcinomas, the frequency of loss, as detected with the probe pYNZ22, was lower than the frequency of loss detected with the probe pBHP53, although not significantly so. When both these probes were informative, every loss detected with the probe pYNZ22 was also detected with pBHP53. These findings argue against tumour suppressor gene(s) located distal to the TP53 gene on chromosome arm 17p involved in colorectal carcinogenesis. Furthermore, these data clearly show that loss detected by either of these probes can be regarded as loss of the TP53 gene.

Thirty-four per cent of the carcinomas had loss on 17q. Loss on 17q was strongly associated with loss of the TP53 gene, suggesting that loss on 17q is frequently part of a more extensive deletion that also comprises the TP53 gene, most likely a part of a whole chromosome loss. The tumours with loss on 17q not involving the TP53 gene constituted 6% of the tumours. This low frequency most likely reflects that loss on 17q is unspecific in these tumours. However, we cannot rule out that among the tumors not showing allele loss on 17q, there may be tumours having abnormalities at a more proximal site of this chromosome arm.

In general, allele amplification may indicate a specific genetic change contributing in the carcinogenesis, or a compensatory mechanism as response to a specific event, as for instance loss of chromosomal segments (Cavenee *et al.*, 1983). Fifteen per cent of the tumours studied had amplification on 17q, and a net gain of DNA sequences on 17q was seen in the majority of these tumours. A gain of chromosome arm 17q in tumours with loss on 17p has earlier been found in colorectal carcinomas by cytogenetic analysis (Muleris *et al.*, 1985, 1988). In our study, the amplification on 17q was neither associated with loss on 17p, nor with loss on the other 17q arm. This is in contrast to a previous report using Southern analysis, demonstrating that gain of one 17q allele was always followed by loss of the other allele (Fearon *et al.*, 1987). Amplification on 17p, found in a small proportion of the tumours (8%), always occurred in combination with loss on the other 17p arm. This is in agreement with earlier reports, demonstrating that monosomy is an unstable condition, and single chromosomes tends to be duplicated after loss has occurred of the other homologue chromosome (Eves *et al.*, 1983; Cavenee *et al.*, 1983). However, since amplification on 17p was associated with loss on the homologue chromosome arm, whereas amplification on 17q was not, different chromosomal mechanisms associated with monosomy of 17p and of 17q may be indicated. The relatively high frequency of amplification on 17q addresses the possibility that amplification on 17q is a specific and not just a random chromosomal event in colorectal carcinomas. This question, however, needs further clarification.

In summary, loss of the TP53 gene was found in the majority of the colorectal carcinomas, and was most frequently part of a limited, subchromosomal deletion, as oppossed to being part of a whole chromosome loss. Furthermore, we found no evidence for any additional tumour suppressor gene on chromosome 17 involved in colorectal carcinogenesis. We therefore conclude that loss of the TP53 gene, as detected by Southern analysis and the two probes on 17p applied, denotes specific loss of this gene, and furthermore, that this loss is of functional importance for the tumours with this change. To analyse the implications of loss of the TP53 gene in colorectal carcinogenesis, we have studied the relationship between loss of the TP53 gene and clinicopathological variables of established prognostic importance. This analysis is presented in article II (Meling *et al.*, 1992).

The authors are grateful to Drs: J.N. Wiig, O.P. Grüner, O.C. Lunde, E. Schlichting, A. Bakka, E. Trondsen, J. Hognestad, Ø. Havig and A. Bergan for supplementing the tumour samples used in the study, and to Dr B. Høyheim for the pBHP53 probe, and Drs Y. Nakamura and R. White for the probes pYNZ22, pTHH59, pRMU3, pYNZ2, and pYNH24. The authors thank Dr Y. Chen, and Ms Aa. Schjølberg for technical assistance. The study is supported by the Norwegian Cancer Society, A/S Freia Chocolate Factory's Medical Fund, The Medical Innovation Foundation, and the Legacy of Astrid and Birger Thorsted, Oslo, Norway. RAL is a post doctoral fellow of the Norwegian Research Council for Science and Humanities.

### References

ANDERSEN, T.I., GAUSTAD, A., OTTESTAD, L., FARRANTS, G.W., NESLAND, J.M., TVEIT, K.M. & BØRRESEN, A.-L. (1992). Genetic alterations of the tumour suppressor gene regions 3p, 11p, 13q, 17p and 17q in human breast carcinomas. *Genes, Chrom. & Cancer*, **4**, 113–121.

BAKER, S.J., PREISINGER, A.C., JESSUP, J.M., PARASKEVA, C., MARKOWITZ, S., WILLSON, J.K.V., HAMILTON, S. & VOGELSTEIN, B. (1990). p53 gene mutations occur in combination with 17p allelic deletions as late events in colorectal tumorigenesis. *Cancer Res.*, **50**, 7717–7722.

BAKER, S.J., FEARON, E.R., NIGRO, J.M., HAMILTON, S.R., PREISINGER, A.C., JESSUP, J.M., TUINEN, P.V., LEDBETTER, D.H., BARKER, D.F., NAKAMURA, Y., WHITE, R. & VOGELSTEIN, B. (1989). Chromosome 17 deletions and p53 gene mutations in colorectal carcinomas. *Science*, **244**, 217–220.

CAVENEE, W.K., DRYJA, T.P., PHILLIPS, R.A., BENEDICT, W.F., GODBOUT, R., GALLIE, B.L., MURPHREE, A.L., STRONG, L.C. & WHITE, R.L. (1983). Expression of recessive alleles by chromosomal mechanisms in retinoblastoma. *Nature*, **305**, 779–784.

CHEN, L.-C., NEUBAUER, A., KURISU, W., WALDMAN, F.M., LJUNG, B.M., GOODSON, W.W., GOLDMAN, E.S., MOORE, D., BALAZS, M., LIU, E., MAYALL, B.H. & SMITH, H.S. (1991). Loss of heterozygosity on the short arm of chromosome 17 is associated with high proliferative capacity and DNA aneuploidy in primary human breast cancer. *Proc. Natl Acad. Sci. USA*, **88**, 3847–3851.

COLES, C., THOMPSON, A.M., ELDER, P.A., COHEN, B.B., MACKENZIE, I.M., CRANSTON, G., CHETTY, U., MACKAY, J., MACDONALD, M., NAKAMURA, Y., HØYHEIM, B. & STEEL, C.M. (1990). Evidence implicating at least two genes on chromosome 17p in breast carcinogenesis. *Lancet*, **336**, 761–763.

DELATTRE, O., OLSCHWANG, S., LAW, D.J., MELOT, T., REMVIKOS, Y., SALMON, R.J., SASTRE, X., VALIDIRE, P., FEINBERG, A.P. & THOMAS, G. (1989). Multiple genetic alterations in distal and proximal colorectal cancer. *Lancet*, **ii**, 353–355.

DUKES, C.E. (1932). The classification of cancer of the rectum. *J. Pathol. Bacteriol.*, **35**, 323–332.

EVES, E.M. & FARBER, R.A. (1983). Expression of recessive Aprt mutations in mouse CAK cells resulting from chromosome loss and duplication. *Somat. Cell Genet.*, **9**, 771–778.

FEARON, E.R., HAMILTON, S.R. & VOGELSTEIN, B. (1987). Clonal analysis of human colorectal tumors. *Science*, **238**, 193–196.

FEINBERG, A.P. & VOGELSTEIN, B. (1983). A technique for radiolabelling DNA restriction endonuclease fragments to high specific activity. *Analyt. Biochem.*, **132**, 6–13.

HØYHEIM, B., NAKAMURA, Y. & WHITE, R. (1989). A BamH1-polymorphism is detected by a genomic p53-clone (pBHP53). *Nucleic Acids Res.*, **17**, 8898.

HGM11. Eleventh International Workshop on Human Gene Mapping (1991). *Cytogenet. Cell Genet.*, **58**, 690.

KNUDSON, A.G. (1985). Hereditary cancer, oncogenes, and antioncogenes. *Cancer Res.*, **45**, 1437–1443.

KUNCKEL, L.M., SMITH, K.D., BOYER, S.H., BORGAONKAR, D.S., WACHTEL, S.S., MILLER, O.J., BREG, W.R., JONES Jr, H.W. & RARY, J.M. (1977). Analysis of human y-chromosome-specific reiterated DNA in chromosome variants. *Proc. Natl Acad. Sci. USA*, **74**, 1245-1249.

LEVINE, A.J., MOMAND, J. & FINLAY, C.A. (1991). The p53 tumour suppressor gene. *Nature*, **351**, 453-455.

LOTHE, R.A., NAKAMURA, Y., WOODWARD, S., GEDDE-DAHL, T. Jr & WHITE, R. (1988). VNTR (variable number of tandem repeats) markers show loss of chromosome 17p sequences in human colorectal carcinomas. *Cytogenet. Cell. Genet.*, **48**, 167-169.

MELING, G.I., LOTHE, R.A., BØRRESEN, A.-L., HAUGE, S., GRAUE, C., CLAUSEN, O.P.F. & ROGNUM, T.O. (1991). Genetic alterations within the Retinoblastoma locus in colorectal carcinomas. Relation to DNA ploidy pattern studied by flow cytometric analysis. *Br. J. Cancer*, **64**, 475-480.

MELING, G.I., LOTHE, R.A., BØRRESEN, A.-L., GRAUE, C., HAUGE, S., CLAUSEN, O.P.F. & ROGNUM, T.O. (1993). The TP3 tumour suppressor gene in colorectal carcinomas. II. Relation to DNA ploidy pattern and clinicopathological variables. *Br. J. Cancer*, **66**, in press.

MULERIS, M., SALMON, R.J., ZAFRANI, B., GIRODET, J. & DUTRILLAUX, B. (1985). Consistent deficiencies of chromosome 18 and of the short arm of chromosome 17 in eleven cases of human large bowel cancer: a possible recessive determinism. *Ann. Genet.*, **28**, 206-213.

MULERIS, M., SALMON, R.J. & DUTRILLAUX, B. (1988). Existence of two distinct processes of chromosomal evolution in near-diploid colorectal tumours. *Cancer Genet. Cytogenet.*, **32**, 43-50.

MYERS, R., NAKAMURA, Y., BALLARD, L., LEPPERT, M., CONNELL, P.O., LATHROP, G.M., LALOUEL, J.-M. & WHITE, R. (1988). Isolation and mapping of a polymorphic DNA sequence pRMU3 on chromosome 17q (D17S24). *Nucleic Acids Res.*, **16**, 784.

NAKAMURA, Y., GILLILAN, S., O'CONNELL, P., LEPPERT, M., LATHROP, G.M., LALOUEL, J.-M. & WHITE, R. (1987). Isolation and mapping of a polymorphic DNA sequence pYNH24 on chromosome 2 (D2S44). *Nucleic Acids Res.*, **15**, 10073.

NAKAMURA, Y., BALLARD, L., LEPPERT, M., CONNEL, P.O.,, LATHROP, G.M., LALOUEL, J.-M. & WHITE, R. (1988*a*). Isolation and mapping of a polymorphic DNA sequence (pYNZ22) on chromosome 17p (D17S30). *Nucleic Acids Res.*, **16**, 5707.

NAKAMURA, Y., HOLM, T., GILLILAN, S., LEPPERT, M., CONNELL, P.O., LATHROP, G.M., LALOUEL, J.-M. & WHITE, R. (1988*b*). Isolation and mapping of a polymorphic DNA sequence (pTHH59) on chromosome 17q (D17S4). *Nucleic Acids Res.*, **16**, 3598.

NAKAMURA, Y., CULVER, M., SERGEANT, L., LEPPERT, M., CONNEL, P.O., LATHROP, G.M., LALOUEL, J.-M. & WHITE, R. (1988*c*). Isolation and mapping of a polymorphic DNA sequence (pYNZ2) on chromosome 1p (D1S57). *Nucleic Acids Res.*, **16**, 4747.

NIGRO, J.M., BAKER, S.J., PREISINGER, A.C., JESSUP, M., HOSTETTER, R., CLEARY, K., BIGNER, S.H., DAVIDSON, N., BAYLIN, S., DEVILEE, P., GLOVER, T., COLLINS, F.S., WESTON, A., MODALI, R., HARRIS, C.C. & VOGELSTEIN, B. (1989). Mutations in the p53 gene occur in diverse human tumour types. *Nature*, **342**, 705-707.

SATO, T., TANIGAMI, A., YAMAKAWA, K., AKIYAMA, F., KASUMI, F., SAKAMOTO, G. & NAKAMURA, Y. (1990). Allelotype of breast cancer: cumulative allele losses promote tumor progression in primary breast cancer. *Cancer Res.*, **50**, 7184-7189.

SOUTHERN, E.M. (1975). Detection of specific sequences among DNA fragments separated by gel electrophoresis. *J. Mol. Biol.*, **98**, 503-517.

TURNBULL Jr, R.B., KYLE, K., WATSON, F.R. & SPRATT, J. (1967). Cancer of the colon: the influence of the no-touch isolation technic on survival rates. *Ann. Surg.*, **166**, 420-427.

VOGELSTEIN, B., FEARON, E.R., HAMILTON, S.R., KERN, S.E., PREISINGER, A.C., LEPPERT, M., NAKAMURA, Y., WHITE, R., SMITS, A.M.M. & BOSS, J.L. (1988). Genetic alterations during colorectal-tumor development. *N. Engl. J. Med.*, **319**, 525-532.